# Monitoring of Cell Concentration during *Saccharomyces cerevisiae* Culture by a Color Sensor: Optimization of Feature Sensor Using ACO

**DOI:** 10.3390/s19092021

**Published:** 2019-04-30

**Authors:** Hui Jiang, Weidong Xu, Quansheng Chen

**Affiliations:** 1 School of Electrical and Information Engineering, Jiangsu University, Zhenjiang 212013, China; w_d_xu@126.com; 2 School of Food and Biological Engineering, Jiangsu University, Zhenjiang 212013, China; qschen@ujs.edu.cn

**Keywords:** yeast culture, process monitoring, color sensor, optimization, ant colony optimization (ACO)

## Abstract

The odor information produced in *Saccharomyces cerevisiae* culture is one of the important characteristics of yeast growth status. This work innovatively presents the quantitative monitoring of cell concentration during the yeast culture process using a homemade color sensor. First, a color sensor array, which could visually represent the odor changes produced during the yeast culture process, was developed using eleven porphyrins and one pH indicator. Second, odor information of the culture substrate was obtained during the process using the homemade color sensor. Next, color components, which came from different color sensitive spots, were extracted first and then optimized using the ant colony optimization (ACO) algorithm. Finally, the back propagation neural network (BPNN) model was developed using the optimized feature color components for quantitative monitoring of cell concentration. Results demonstrated that BPNN models, which were developed using two color components from FTPPFeCl (component B) and MTPPTE (component B), can obtain better results on the basis of both the comprehensive consideration of the model performance and the economic benefit. In the validation set, the average of determination coefficient $R_{P}^{2}$ was 0.8837 and the variance was 0.0725, while the average of root mean square error of prediction (RMSEP) was 1.0033 and the variance was 0.1452. The overall results sufficiently demonstrate that the optimized sensor array can satisfy the monitoring accuracy and stability of the cell concentration in the process of yeast culture.

## 1. Introduction

*Saccharomyces cerevisiae*, also known as budding yeast or bread yeast, is a gram stain positive bacterium [1]. It is a single-celled fungus organism, spherical or oval in shape and 3–8 μm in size. The bacterial colony is medium in size, flat, smooth, moist, cheesy to light brown, and with a wine-like smell. It has the characteristics of rapid reproduction, short growth cycle, easy to carry out large-scale culture, and rich in protein, vitamins, fat, polysaccharides, and various metabolites together with other physiological active substances. So, it is widely used in the food fermentation industry [2,3,4] and the feed industry [5,6,7,8]. However, the yield of fermentation is closely related to the yeast concentration in the yeast fermentation. Therefore, it is of great significance to realize online and rapid detection of the yeast concentration for understanding and controlling the fermentation process and improving the quality of products.

In the process of yeast culture, traditional chemical analysis is the most commonly used method for the detection of cell concentration [9,10,11]. Although this traditional method can obtain more objective test results, it is time-consuming and requires chemical reagents. In recent years, with the development of modern nondestructive testing technology, near infrared spectroscopy (NIRS) has been innovatively applied for rapid nondestructive monitoring of the process of yeast culture [12,13,14]. NIRS is mainly based on the fact that the vibration of hydrogen-containing groups in the material can generate absorption spectra in the near infrared (NIR) region, and a quantitative model between absorption spectra and material components is established with the help of the chemometrics method, so as to realize the rapid nondestructive testing of target properties. However, in different stages of yeast culture, it will inevitably lead to changes in the composition and odor of the volatile organic compounds (VOCs) produced with the growth of microorganisms and the change of substrate components. Thus, the VOCs produced can be related to the cell concentration in the culture medium, although few studies have been reported so far.

Olfactory sensor technology, also known as electronic nose technology, has been successfully applied in some fields, such as food quality analysis [15,16,17,18], discrimination of biological materials [19,20,21], analysis of smell and smell interaction [22,23,24,25,26], but there are still some problems in the implementation process. For example, electronic nose systems assembled with metal oxide sensors (MOS) are susceptible to ambient temperature and humidity in application, resulting in poor selectivity and low sensitivity to volatile gases [27]. In recent years, olfactory visualization technology based on color sensor array has developed rapidly. The technology has a strong specificity, high sensitivity and is not affected by the humidity characteristics because the color sensitive material used has a broad spectrum. Olfactory visualization technology has been successfully used in gas detection [26] and for liquid foodstuffs, such as coffee [28,29,30], wine [31,32], and vinegar [33,34,35,36]. Therefore, from the technical point of view, this technology is also capable of achieving quantitative analysis of the yeast culture process.

In this study, a color sensor array was developed to “visualize” odor change information during the yeast culture process, so as to realize change monitoring of cell concentration in the process of yeast culture. The detailed contents of the research are as follows: (1) constructing a homemade color sensor array based on optimized color sensitive materials; (2) visualizing the representation of odor information from yeast culture media and extracting color features; and (3) optimizing feature sensors using the ACO algorithm and monitoring of cell concentration during the yeast culture process based on the BPNN model.

## 2. Materials and Methods

### 2.1. Sample Preparation

First, 1 mL of activated *Saccharomyces cerevisiae* was inoculated in 100 mL sterile wort medium. The temperature of the incubator was set to 30 °C with a speed at 150 r/min and continuous culturing for 24 h. Secondly, twenty 100 mL sterilized tripod bottles were prepared and numbered in the order of 1 to 20 to prevent the possible problem of bacterial contamination in each sampling. Then 25 mL of liquid seed culture medium was loaded into each numbered tripod, and the number 1 tripod was set as the control group, and the number 2 to 19 tripod was set as the treatment group. Sampling operations were performed at 19 time points (i.e., 0, 4, 8, 12, 16, 20, 24, 28, 32, 36, 40, 44, 48, 52, 56, 60, 64, 68, and 72 h). Then, 0.1 mL of culture solution was loaded in a triangular flask with liquid seed medium, with two triangular flasks from the control group and 0 h were immediately put into a 4 °C refrigerator for storage after shaking. The remaining 18 triangular flasks, which had been vaccinated with culture solution, were set to a concussion incubator at 28 °C with 150 r/min oscillation. Finally, one triangular flask was taken out every 4 h for measurement of the optical density (OD) value and data acquisition of the color sensor.

In this study, a total of 8 batch experiments were carried out according to the above experimental materials and methods. A total of 152 samples was finally obtained as 19 samples could be obtained in each batch of yeast culture.

### 2.2. Constructing the Color Sensitive Sensor Array

According to the preliminary experimental results, eleven porphyrins (Sigma-Aldrich Co., Ltd., St. Louis, MO, USA) and one hydrophobic pH indicator (Sinopharm Chemical Reagent Co., Ltd., Shanghai, China) were finally determined for color sensor manufacturing. The color sensitive materials selected were as follows:

(1) 5,10,15,20-Tetraphenyl-21*H*,23*H*-porphine (TPP);
(2) 5,10,15,20-Tetraphenyl-21*H*,23*H*-porphine manganese (III) chloride (TPPMnCl);
(3) 5,10,15,20-Tetrakis(4-methoxyphenyl)-21*H*,23*H*-porphine iron (III) chloride (FTPPFeCl)
(4) 5,10,15,20-Tetraphenyl-21*H*,23*H*-porphine iron (III) chloride (TPPFeCl)
(5) 5,10,15,20-Tetraphenyl-21*H*,23*H*-porphine copper (II) (TPPCu)
(6) 5,10,15,20-Tetrakis(4-methoxyhenyl)-21*H*,23H-porphine cobalt (II) (FTPPCo)
(7) 5,10,15,20-Tetraphenyl-21*H*,23*H*-porphine zinc (TPPZn)
(8) meso-Tetra(4-carboxyphenyl)-porphine tetramethyl ester (MTPPTE)
(9) meso-Tetraphenyl porphyrin-Ni(II) chlorin (MTPPNiCl)
(10) 2,3,7,8,12,13,17,18-Octaethyl-21*H*,23*H*-porphine nickel (II) (OEPPNi)
(11) meso-Tetraphenyl porphyrin (chlorin fee) (MTPP)
(12) Bromothymol blue (BTB)

The color sensor array (4 × 3) was prepared using the above materials, and the process of the color sensor fabrication was as follows: (1) For each color sensitive material, first, 10 mg of the color sensitive material was weighed accurately and dissolved with dichloromethane, and put it into a 5 mL volumetric flask. Then, in order to speed up dissolution, the solution was treated with ultrasound for 15 min to obtain solutions with a concentration of 2 mg/mL. (2) Then 1 μL of the solution was extracted using a capillary tube (100×0.3 mm) and the point sample was carried out by an array template auxiliary point sample on the reversed-phase silica gel plate. (3) The color sensor array was obtained when the color sensitive material volatilized to a stable state on the substrate material and then, stored in a single sealed sample bag for later use.

### 2.3. Measurement of OD Values

The spectrophotometer (UV-2204PC) was turned on to preheat for 20 min, and the wavelength of the spectrophotometer was set to 600 nm. Adjustment the 0 position of the instrument, and the light transmittance to 100% were carried out. The determination was carried out after repeating the adjustment several times to stabilize the instrument. When the OD values of all samples were measured, a sterile liquid seed culture medium was used as the blank control. The OD value of each sample was detected three times, and the average result of the three measurements was recorded as the OD value of the sample.

In the determination of yeast concentration by the spectrophotometer, the OD value of measured samples should be between 0.1 and 0.65 in order to ensure a good linear relationship between the OD value and the cell concentration. However, the OD value of the measured sample will exceed this interval if the mass concentration of the yeast suspension is larger. Therefore, a suspension solution with a high mass concentration should be diluted appropriately to ensure that the measured OD value is between 0.1 and 0.65, and the real OD value of the sample will then be equal to the dilution multiple times the measured OD value.

### 2.4. Data Acquisition and Preprocessing

**Figure 1:**
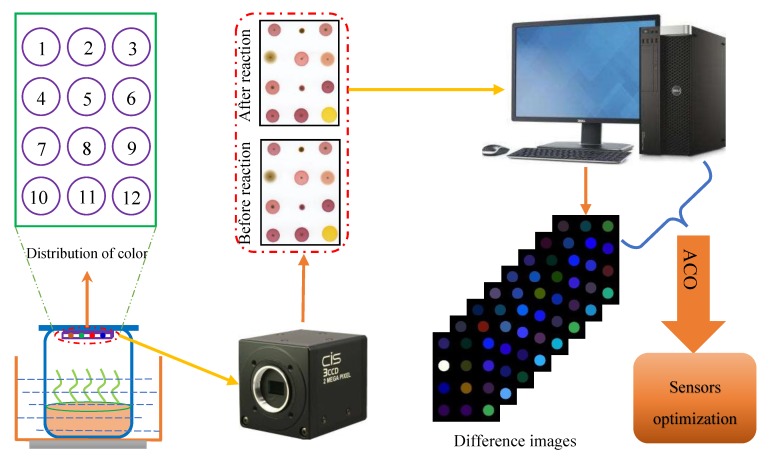
Schematic diagram of the color sensitive sensor array for monitoring of the yeast culture process and sensor optimization.

Figure 1 showed the schematic diagram of the color sensor technique for monitoring the yeast culture process. The specific workflow is as follows: First, the pre-image of the color sensor was acquired by using a 3CCD camera. Second, 10 mL of the culture substrate sample was added into a 50 mL beaker, and the color sensor was fixed on the cover of a homemade plastic film. The color sensor was fully reacted with the odor components of the culture substrate for 10 min in a water bath at 55 °C. Finally, the image after reaction of the color sensor was also obtained by using a 3CCD camera.

In this study the images before and after the reaction obtained by the 3CCD camera were first preprocessed using median filtering, threshold binarization, and morphological processing, and the center of each color sensitive spot on the sensor array was marked as the center of the circle. The circle with a radius of 15 pixels for each color sensitive spot was extracted as the region of interest (ROI) of each color sensitive spot. Then, the gray mean values of the red (R), green (G), and blue (B) components were extracted in the ROI area of each color sensitive spot before and after the reaction, and the gray mean values were subtracted to obtain the feature differences of R, G, and B components before and after the reaction (i.e., ΔR, ΔG, and ΔB). Finally, the feature graph was generated by normalizing the absolute values of ΔR, ΔG, ΔB and subsequently superimposing the grayscale image. Since three color components of ΔR, ΔG, and ΔB can be obtained from each color sensitive spot, 36 color features could be extracted from each sample for subsequent multivariate analysis.

### 2.5. Data Analyses Methods

#### 2.5.1. Back Propagation Neural Network

Back propagation neural network (BPNN) is the most basic neural network. Its output results adopt forward propagation, and the error adopts reverse propagation [37]. The back transmission of error layer by layer is to transmit the output error from the hidden layer to the input layer, and the error signal obtained from each layer is then used as the basis to adjust the weights of each neuron. The error decreases along the gradient direction by adjusting the connection strength of the input node and hidden layer node and the connection strength and threshold value of the hidden layer node and output node. After repeated learning and training, the network parameters (weight and threshold) corresponding to the minimum error are determined, and the training is stopped. The steps of the BPNN algorithm are as follows:

Step 1: Network initialization;

Step 2: Calculating the output of the hidden layer;

Assume both hidden layer and output layer neurons adopt the Sigmoid function, then

(1) $$f\left(x\right)=\frac{1}{1+e^{-x}}$$

(2) $$H_{j}=f\left(\overset{n}{\underset{i=1}{\sum }}w_{ij}x_{i}-\theta_{j}\right)$$

where *x* represents the input variables, and the *x_i_* is the *i*th input variable. *n* is the number of hidden layer nodes. *H_j_*, which is also the input variable of the output layer node, the output result of the *j*th hidden layer node. *w_ij_* represents the weight of the *i*th input variable connected to the *j*th hidden layer node, and $\theta_{j}$ is the threshold value on the *j*th hidden layer node.

Step 3: Calculating the output of the output layer;

(3) $$O_{k}=\sum_{j=1}^{q}H_{j}w_{jk}-b_{k}$$

where $O_{k}$ is the output result of the *k*th output layer node. *q* is the number of output layer nodes. *w_jk_* is the weight of the *j*th hidden layer output variable connected to the *k*th output layer node, and *b_k_* is the threshold value of the *k*th node in the output layer.

Step 4: Calculating errors; 

The error $e_{k}$ and the mean square error $E_{k}$ are calculated according to the actual output and the expected output.

(4) $$e_{k}=Y_{k}-Q_{k}$$

(5) $$E_{k}=\frac{1}{2}\overset{l}{\underset{j=1}{\sum }}{\left(Q_{k}-Y_{k}\right)}^{2}$$

where $Y_{k}$ is the actual value of the target attribute from the *k*th sample, and $Q_{k}$ is the predicted result of the target attribute from the *k*th sample.

Step 5: Updating weight;

(6) $$w_{ih}=w_{ih}+\eta H_{j}\left(1-H_{j}\right)x_{i}\overset{n}{\underset{k=1}{\sum }}w_{ih}e_{k}$$

(7) $$w_{hj}=w_{hj}+\eta H_{j}e_{k}$$

where η is the learning rate. *h* represents the number of iterations, and *w_ih_* and *w_hj_* are the updated connection weights of the input layer to the hidden layer and the hidden layer to the output layer, respectively.

Step 6: Updating threshold;

(8) $$\theta_{j}=\theta_{j}+\eta H_{j}\left(1-H_{j}\right)x_{i}\overset{l}{\underset{k=1}{\sum }}w_{jk}e_{k}$$

(9) $$\gamma_{h}=\gamma_{h}+e_{h}$$

where $e_{h}$ represents the network error after the end of the *h*th iteration, and $\gamma_{h}$ is the updated error before the next iteration.

Step 7: According to the output, if the error is within the allowable range, and the algorithm ends; otherwise, skip to Step 2.

In this study, BPNN was employed to establish a regression model between the color sensor features and the OD values of all samples. The parameters of the BPNN network were set as follows: the number of neurons in the hidden layer was set to 10, the learning rate factor and momentum factor were set to 0.1, the initial weight was set to 0.3, and the scale function was set as “trainlm” function. The permitted training error was set to 0.01; and the maximal times of training were set to 1000.

#### 2.5.2. Ant Colony Optimization Algorithm

The ant colony optimization (ACO) algorithm is an intelligent optimization evolutionary algorithm based on parametric probability distribution, and the parameters of the model are pheromones released by individual ants [38]. In fact, ACO is an intelligent optimization algorithm with pheromone as its core.

The potential solutions of common pheromone model optimization algorithms are all calculated based on one of the probability distribution models. The feasible solution of this model is calculated using the iterative optimization method to further update its own parameters. The search scope of the model gets closer to the optimal solution space, and then searches for a better solution in the optimal solution cluster. The optimal solution can only be effective if the high-quality solution can meet the actual problem and meet certain requirements. The ACO algorithm finds the potential relationship between the information of the solutions constitution and quality by the optimization iteration loop to further construct a high-quality solution that meets the requirements. The following three Formulas (10), (11), and (12) describe the ACO algorithm.

(10) $$p_{ij}^{k}=\frac{\tau_{ij}^{\alpha }\eta_{ij}^{\beta }}{\sum_{j\in \Lambda }\tau_{ij}^{\alpha }\eta_{ij}^{\beta }}$$

(11) $$\tau_{ij}\left(n+1\right)=\rho \times \tau_{ij}\left(n\right)+\overset{m}{\underset{k=1}{\sum }}\Delta \tau_{ij}^{k}$$

(12) $$\Delta \tau_{ij}^{k}=\frac{Q}{L_{k}}$$

where *k* is the individual number of the ant colony; *i* is the current position of the individual ant colony, and *j* is the position that the individual ant colony can choose. Formula (10) is used to calculate the probability that ant *k* at position *i* chooses position *j*. *Λ* is a set of optional locations at position *i* up and down; $\tau_{ij}$ is the pheromone concentration between location *i* and *j*; $\eta_{ij}$ is the visibility of position *i* and *j*; α and β are the weights of pheromone concentration and visibility, respectively; which represent the degree of influence to choose the position for the ants. Formula (11) is to calculate the variation of pheromone concentration between position *i* and *j*, mainly including natural evaporation and ant release; ρ is the pheromone evaporation coefficient of the path. Formula (12) is the ant cycle system model of the pheromone quantity secreted by individual ants on the road, where *Q* is the total amount of pheromone released by individual ants. *L*_k_ in the ant cycle system model is the distance traveled by the ant, and the ant determines the amount of pheromone released according to the length of the globally optimal road. The ant cycle system model is adopted to calculate the pheromone quantity secreted by ants on the road, which can ensure the convergence and stability of the ACO algorithm. Normally, the smaller the distance, the more pheromones the ants leave behind.

In this study, the ACO algorithm was used to optimize the color feature components of the color sensor according to the mean deviation between the measured value and the predicted value of the BPNN model in the calibration and validation sets. Therefore, the objective function (i.e., BestCost) of the ACO algorithm was defined as follows:

(13) $$\mathrm{BestCost}=0.75\times \frac{\sum_{i=1}^{n_{train}}e_{i}}{n_{train}}+0.25\times \frac{\sum_{j=1}^{n_{test}}e_{j}}{n_{test}}$$

where *n*_train_ is the number of samples in the calibration set, and *e*_i_ represents the deviation between the measured value of each sample and the predicted value of the BPNN model in the calibration set and *n*_test_ is the number of samples in the validation set, while *e*_j_ represents the deviation between the measured value of each sample and the predicted value of BPNN model in the validation set. In addition, the parameters of the ACO algorithm were set as follows: the population size (i.e., number of ants) was set to 20, and the maximum number of iterations was set to 100. The initial pheromone concentration τ was set to 1, and the weights of the pheromone concentration (α and β) were both set to 1. The ρ of the pheromone evaporation coefficient was set to 0.05.

### 2.6. Software

All the algorithms were implemented in Matlab R2018a (Mathworks, Natick, MA, USA) under Windows 10 in data processing. The feature selection algorithm, which combines ACO and BPNN used in this study, was developed and written by our team.

## 3. Results

### 3.1. Reference Measurement of OD Values

Before the model calibration, all 152 culture samples were divided into two subsets. One was the calibration set, and the other was the validation set. In this study, a total of eight batches of yeast culture experiments were conducted. Thus, samples from the previous six batches of the culture experiments, which contained 114 samples, were included in the calibration set, and the remaining 38 samples from the latter two batches were included in the validation set. Table 1 shows the statistical results of OD values in the calibration and validation sets. As can be seen from Table 1, the sample division is reasonable based on statistical theory, since the calibration set covers the range of the target attributes of the validation set.

### 3.2. Results of Sensor Responses

**Figure 2:**
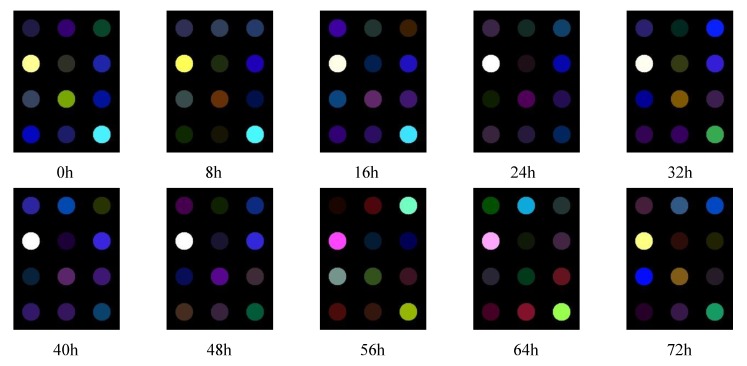
Chromatic difference images for samples obtained with interval 8 h during the yeast culture process.

The homemade color sensor array can transform the odor information of yeast media into visible image information, which can make the results presented more intuitive and visual. Figure 2 shows the difference images of the substrate samples of the various culture stages. As can be seen from Figure 2, the color changes are different from the reaction difference spots of the different color sensitive chemical dyes on the black background. Thus, yeast fermentation broths at different stages of culture have their own distinctive feature difference image. It was suggested that the developed color sensor array had higher sensitivity to odor components of the different stages of the samples. Therefore, it is feasible to use color sensor technology to realize the monitoring of cell concentration during the process of yeast culture. However, it is clear that some spots of the chemical dyes are similar in color, and some are almost identical. This indicates that different color sensitive materials have cross-sensitivity to the odor of yeast media, which leads to certain redundant information between the images of the spots. Therefore, it is necessary to further optimize the features of the preselected sensors in order to reduce the cost and complexity of manufacturing the color sensor array.

### 3.3. Results of Sensor Optimization Using ACO

**Figure 3:**
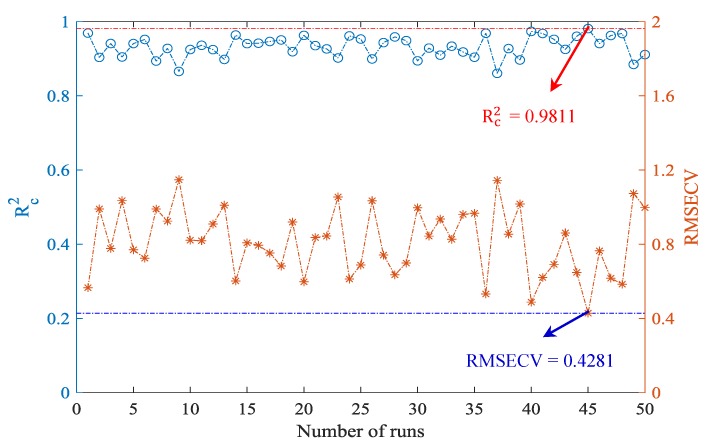
Statistics results of $R_{c}^{2}$ and root mean square error of cross-validation (RMSECV) values in the calibration set after 50 runs of the ACO algorithm.

**Figure 4:**
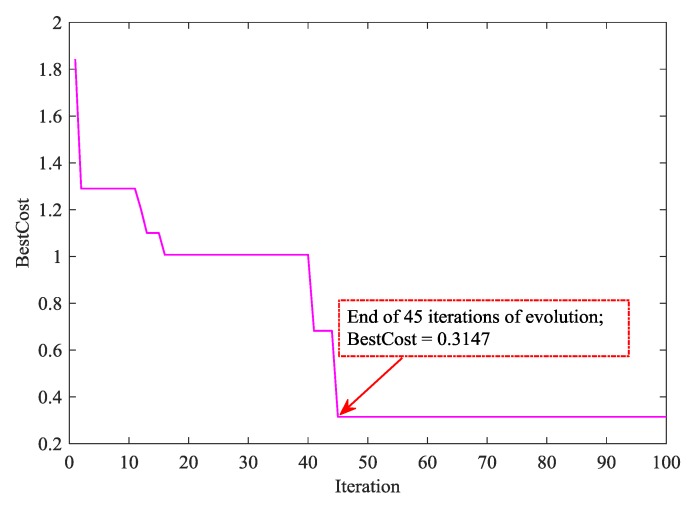
The convergence curve of the optimal model during 50 runs of the ACO algorithm.

ACO has a certain randomness during the initialization of the algorithm. Therefore, in this study, 50 independent runs of the ACO algorithm were carried out under the same parameter setting in order to eliminate the influence of randomness. Then, the results after 50 independent runs were statistically analyzed. Figure 3 shows the statistical results of the determination coefficient $R_{c}^{2}$ and root mean square error of cross-validation (RMSECV) values in the calibration set after 50 runs of the ACO algorithm. As can be seen from Figure 3, the average of $R_{c}^{2}$ value was 0.9321 and the variance was 0.0287. The average of RMSECV was 0.8126 and the variance was 0.1780. The above results suggested that the BPNN model achieved comparatively ideal results in accuracy and stability. It is worth mentioning that the $R_{c}^{2}$ value of the BPNN model reached the highest value of 0.9811, while the RMSECV value was only 0.4281 after ACO optimization in the calibration set. Figure 4 shows the convergence curve of the ACO algorithm with the best results during the 50 runs. From Figure 4, the ACO algorithm converged after 45 iterations, and its optimal objective function value was 0.3147.

**Figure 5:**
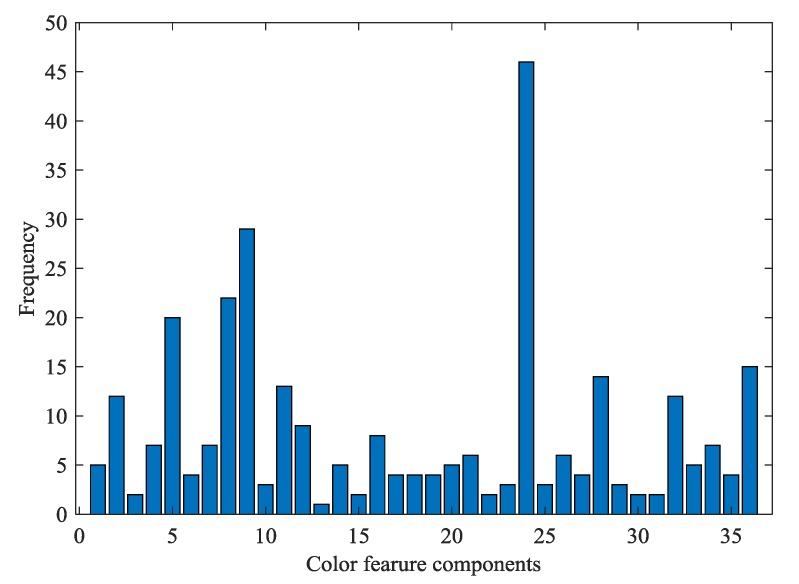
Cumulative frequency of each color component selected after 50 runs of the ACO algorithm.

Figure 5 showed the frequency of all color components selected after 50 runs of the ACO algorithm. As can be seen from Figure 5, there were two color variables that were selected up to 25 times in the 50 runs of the algorithm, and they came from the color sensitive materials FTPPFeCl (component B) and MTPPTE (component B), respectively. In the 50 runs of the ACO algorithm, four color components were selected more than 20 times, and came from the color sensitive materials TPPMnCl (component B), FTPPFeCl (component G and B), and MTPPTE (component B). There were also five color components with the selected frequency achieving 15 times, which came from the color sensitive materials TPPMnCl (component B), FTPPFeCl (component G and B), MTPPTE (component B), and BTB (component B), respectively.

Table 2 shows the statistical results of 50 independent runs of the BPNN model based on the above three cases of selected variables. As can be seen from Table 2, the BPNN models based on four and five characteristic color components have similar performances in the calibration set. Both are superior to the BPNN models on the basis of two characteristic color components. Although the model performance based on two color components is weaker than those based on four or five color components, the former requires only two color sensitive materials, while the latter requires four or five. As we all know, the price of color sensitive materials is relatively expensive especially as the performance and stability of these models show little difference in predicting the independent samples in a validation set. It could be considered worth sacrificing a little precision in the model in order to obtain more economical sensor preparation. From Table 2, through the optimization of the color sensitive sensor, it can be determined that the BPNN model based on the feature color components of materials FTPPFeCl and MTPPTE can well realize process monitoring of the odor information of the yeast culture, while taking into account the economic benefits of sensor preparation.

## 4. Conclusions

In this study, a novel color sensor was designed to realize the monitoring of cell concentration during yeast culture. An ACO algorithm was employed to optimize the features of the primary color sensitive materials. The results of research show that two, four, and five color feature components optimized by the ACO algorithm were used to construct the BPNN models, which all could effectively realize the process monitoring of odor information during yeast culture, while the results were similar especially in the predictive process. Therefore, FTPPFeCl and MTPPTE were finally selected and could be used to prepare relatively inexpensive sensor arrays, which can meet the needs of accuracy and stability of cell concentration monitoring during the yeast culture process. It can be inferred that the sensor array will have promising applications for monitoring of cell concentration in application in industrial processes.

## Figures and Tables

**Table 1:** Reference measurement results of optical density (OD) values in the calibration and validation sets.

Subsets	S. N. ^a^	Range (%)	Mean	S. D. ^b^
Calibration set	114	0.001–9.120	5.7080	3.1233
Validation set	38	0.001–8.900	5.6734	3.2858

S. N. ^a^, sample number; S. D. ^b^, standard deviation

**Table 2:** Statistical results from 50 runs of BPNN models based on different color components.

Model	Number of Color Components	Calibration Set		Validation Set	
		$R_{c}^{2}$	RMSECV	$R_{p}^{2}$	RMSEP
Case 1	2	0.9362 ± 0.0272	0.8187 ± 0.1580	0.8837 ± 0.0725	1.0033 ± 0.1452
Case 2	4	0.9638 ± 0.0221	0.6018 ± 0.1531	0.8864 ± 0.0848	1.0015 ± 0.1513
Case 3	5	0.9690 ± 0.0254	0.5656 ± 0.1625	0.8965 ± 0.0786	0.9541 ± 0.1526

Note: Case 1, features FTPPFeCl (component B) and MTPPTE (component B); Case 2, features TPPMnCl (component B), FTPPFeCl (component G and B) and MTPPTE (component B); Case 3, features TPPMnCl (component B), FTPPFeCl (component G and B), MTPPTE (component B), and BTB (component B).
